# RF sensing enabled tracking of human facial expressions using machine learning algorithms

**DOI:** 10.1038/s41598-024-75909-w

**Published:** 2024-11-13

**Authors:** Hira Hameed, Mostafa Elsayed, Jaspreet Kaur, Muhammad Usman, Chong Tang, Nour Ghadban, Julien Le Kernec, Amir Hussain, Muhammad Imran, Qammer H. Abbasi

**Affiliations:** 1 University of Glasgow, James Watt School of Engineering, G12 8QQ Glasgow, UK; 2 School of Computing, Engineering and Built Environment, Glasgow Caledonian University, Glasgow, G4 0BA UK; 3 School of Computing, Edinburgh Napier University, Scotland, UK; 4 Artificial Intelligence Research Center (AIRC), Ajman University, Ajman, UAE

**Keywords:** Health care, Engineering

## Abstract

Automatic analysis of facial expressions has emerged as a prominent research area in the past decade. Facial expressions serve as crucial indicators for understanding human behavior, enabling the identification and assessment of positive and negative emotions. Moreover, facial expressions provide insights into various aspects of mental activities, social connections, and physiological information. Currently, most facial expression detection systems rely on cameras and wearable devices. However, these methods have drawbacks, including privacy concerns, issues with poor lighting and line of sight blockage, difficulties in training with longer video sequences, computational complexities, and disruptions to daily routines. To address these challenges, this study proposes a novel and privacy-preserving human behavior recognition system that utilizes Frequency Modulated Continuous Wave (FMCW) radar combined with Machine Learning (ML) techniques for classifying facial expressions. Specifically, the study focuses on five common facial expressions: Happy, Sad, Fear, Surprise, and Neutral. The recorded data is obtained in the form of a Micro-Doppler signal, and state-of-the-art ML models such as Super Learner, Linear Discriminant Analysis, Random Forest, K-Nearest Neighbor, Long Short-Term Memory, and Logistic Regression are employed to extract relevant features. These extracted features from the radar data are then fed into ML models for classification. The results show a highly promising classification accuracy of 91%. The future applications of the proposed work will lead to advancements in technology, healthcare, security, and communication, thereby improving overall human well-being and societal functioning.

## Introduction

Facial recognition technology has witnessed substantial advancements in recent years, finding widespread applications across various domains. These applications include healthcare and mental health, human-robot interaction, biometric identification, human-computer interaction, security and surveillance, as well as entertainment and gaming. It is also treated as a nonverbal sign used by people to convey emotions, intentions, and social signals^1^. The paper^2^ presents that understanding and monitoring facial expressions has piqued the interest of researchers in a variety of fields, including psychology, neuroscience, and computer vision.

Face expressions are ubiquitous and may be used to communicate effectively across cultures and languages^3^. They are essential in social interactions because they let humans express emotions such as happiness, sadness, anger, fear, surprise, disgust, and contempt^4^. Facial expressions are caused by the coordinated movement of facial muscles, which results in recognized and interpretable patterns by human observers^5^. Affective computing, human-computer interaction, healthcare, and social robotics all benefit from the capacity to reliably monitor and comprehend facial emotions^6^.

Camera-based face expression monitoring systems are substantial. These systems rely on cameras to capture facial photos or videos, which are then analyzed to determine facial expressions. Many studies have presented several algorithms to effectively recognize and classify face expressions, including classic computer vision approaches and deep learning techniques^7–9^. In this paper^10^, the framework integrates edge computing and optimized mobile network design to enable efficient and real-time facial expression recognition on mobile devices. The author^11^ presented a system that utilizes PTZ cameras and the Faster R-CNN algorithm for facial expression analysis and student engagement recognition. It described the system’s architecture, training process, and experimental results, highlighting its potential contributions to the field of educational technology and classroom management. Camera-based systems provide high-resolution facial images, allowing for a thorough analysis of facial traits. Camera-based systems have significant drawbacks, for example, require subjects to be inside the field of view and may be affected by lighting conditions, privacy issues, occlusions, and the requirement for direct line-of-sight^12^. These constraints can limit the usability and accuracy of camera-based solutions, especially in uncontrolled or real-world environments.

Wearable gadgets such as smart glasses, headphones, and wristbands have been investigated as alternate platforms for monitoring facial expressions^13^. Sensors such as accelerometers, gyroscopes, and electroencephalography (EEG) sensors are used in these devices to gather physiological data linked with face muscle movements^14^. Researchers built algorithms to infer face expressions by examining the collected data^15^. Wearable technology provides the benefit of portability as well as the capacity to observe face expressions in real-time throughout regular activities. They do, however, have restrictions^16^. Wearable technologies can be obtrusive, inconvenient, and deliver inaccurate or incomplete data. Furthermore, sensor installation and calibration might be difficult, resulting in significant mistakes in face expression interpretation^17^. A major limitation of wearable-based facial expression recognition is its interference with daily routines, as individuals are required to constantly carry and wear the devices.

In contrast, radio frequency (RF) facial expression sensors present a promising solution to meet the requirements of next-generation technologies. These sensors utilize RF sensing and machine learning (ML) techniques to accurately detect and recognize facial expressions, offering valuable cues that can benefit a wide range of applications. The paper discusses the design considerations, circuit architecture, and implementation details of the IF waveform generation and acquisition circuits. The proposed circuits are evaluated and validated through experimental results, demonstrating their suitability for radar system applications^18^. These approaches collect and analyse facial muscle movements and electromagnetic scattering patterns induced by facial expressions using RF signals^19^. The author^20^ discusses RF sensing technologies for monitoring various health-related parameters such as respiration, heart rate, activity level, and sleep quality. They explore different RF-based sensing techniques, including Doppler radar, RFID, and Wi-Fi sensing, highlighting their respective advantages and limitations.. RF signals can pass through impediments like clothing and walls, enabling continuous monitoring in uncontrolled conditions^21,22^. The author^23^ used wireless signals, specifically the channel state information (CSI) gathered from Wi-Fi devices, to detect the subtle alterations in the environment resulting from human emotional states. The classification accuracy achieved for emotions was 71.67%. The paper presents the architecture and working principle of the WiFace system, which involves capturing and analyzing the variations in Wi-Fi signals reflected off a person’s face. A total of six activities were performed by the subject Happy, Fearful, Surprised, Happily surprised, Angrily surprised, and Fearfully surprised^24^. Table 1 shows the comparison between vision-based and sensor-based technology for facial expression recognition.

**Table 1:** Comparison of vision-based and sensor-based emotion recognition with our work.

References	Technology used	Activity used	AI model	Accuracy (%)
^25^	Vision-based	Facial expression	ResNet-50	95.39 ± 1.41
^10^	Vision-based	Facial expression	DNN	85.57
^26^	Vision-based	Facial expression	3D-CNN and ConvLSTM	98.83
^27^	Vision-based	Facial expression	DBN	96.25
^28^	Sensor-based	Facial muscle movements	Cross-domain transfer learning	80.75
^29^	Sensor-based	Emotion recognition	Random Forest	60–70%
^30^	Sensor-based	Respiration and heart rate signals with emotion recognition	CNN and GRU	84.5% and 74.25%
^31^	Sensor-based	Emotion recognition	Neural network	80.59
Our	Sensor-based	Emotion recognition	LSTM	91.0%

The following presents the main contributions of our research work in the field:

- RF technology enhances facial expression applications through non-intrusive, privacy-preserving monitoring, and remains effective even in poor lighting conditions, ensuring accurate detection across various environments.
- We proposed a unique RF sensing-based facial expression monitoring system that integrates powerful machine learning algorithms for accurate facial expression recognition and is applicable for different applications such as healthcare and mental health, human-robot interaction, biometric identification, human-computer interaction, security and surveillance, as well as entertainment and gaming.
- A dataset comprising 1,000 samples was gathered, encompassing five distinct facial expressions because these are the most common and part of normal routines. The data collection took place at a consistent distance of 1.50 meters from the target. To ensure variability and inclusiveness, four participants were involved in the data collection process. These participants consisted of two males and two females within the age range of 20 to 40 years, all belonging to different ethnic backgrounds, including Chinese, Asian, and English.
- We used extensive experiments and comparisons with existing camera-based and wearable device-based technologies to assess the performance and usefulness of the proposed RF-based system, demonstrating the benefits and prospective uses of RF sensing in facial expression monitoring.
- In this study, we have presented the experimental results of several advanced machine learning models applied to our benchmark dataset. These findings provide valuable insights and can serve as a fundamental reference for future research in the domain of facial expression detection.

**Fig. 1:**
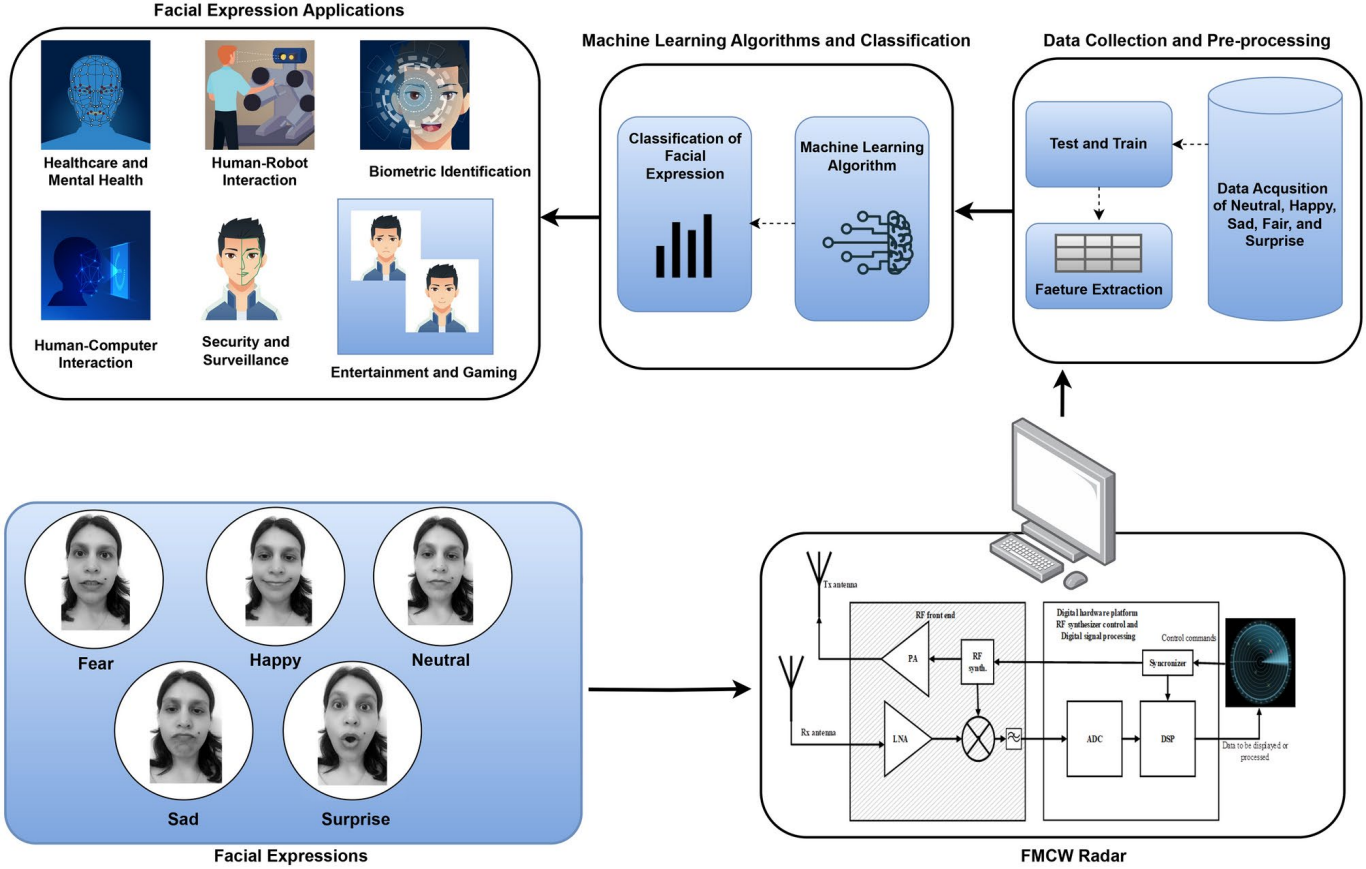
The overall flow diagram of proposed facial expressions system.

This research introduces innovative facial expression gestures using radar-sensor and micro-doppler signatures. The study focuses on five distinct gestures: Neutral, Happy, Sad, Fear, and Surprise. Experimental data is collected using an FMCW radar, and the recorded data is represented as a Micro-Doppler signal. The classification accuracy achieved is 91.0%. The potential applications of this technology are illustrated in Fig. 1. Detailed information regarding the setup, data collection process, machine learning algorithms, and experimental results are provided in the subsequent sections.

## Methodology

**Fig. 2:**
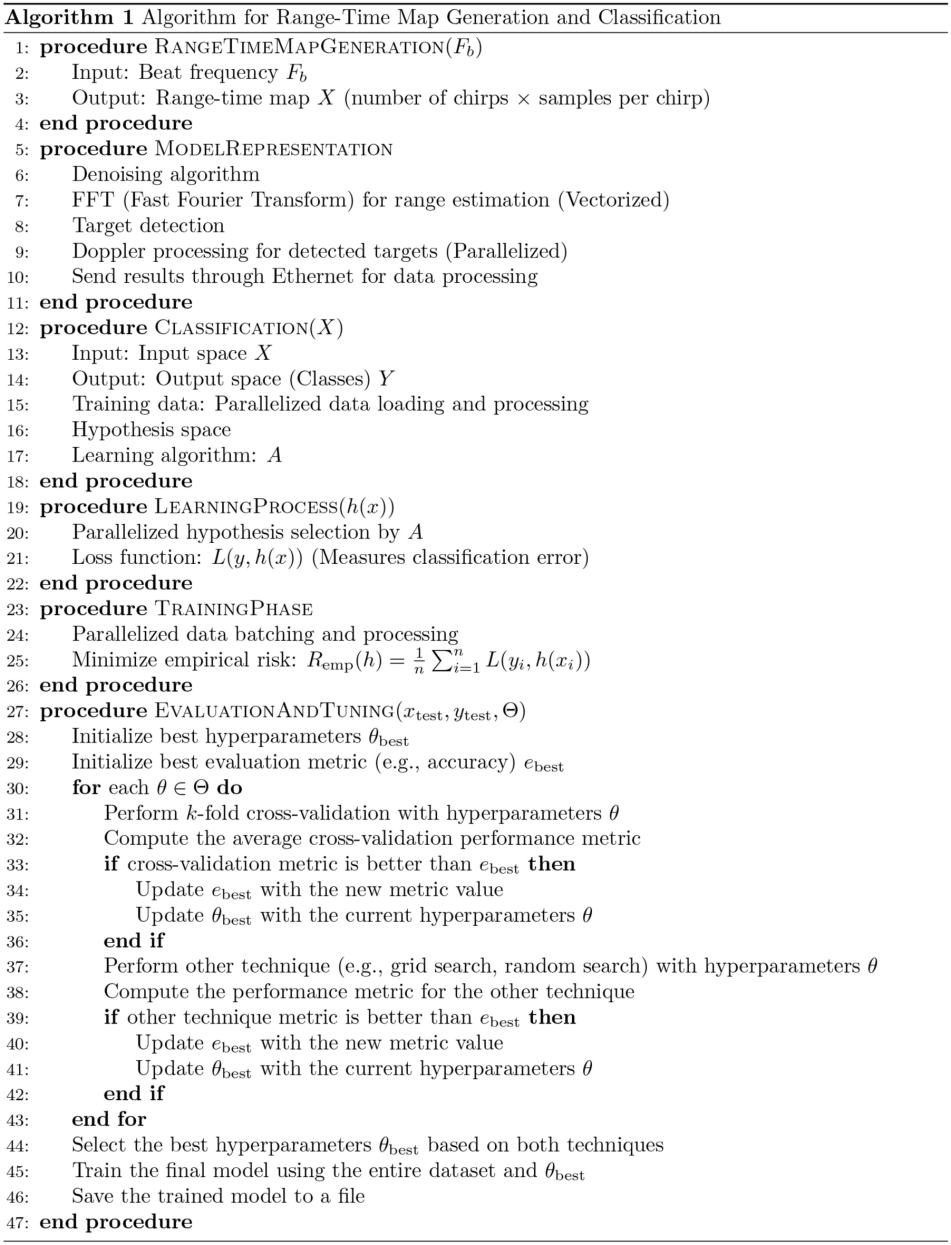
Pseudo code for the proposed facial expression systems.

The methodology used in this study is depicted in Fig. 1 as a block diagram. The framework comprises three main steps. Firstly, diverse facial expression datasets were collected, constructed, and annotated using FMCW radar. Subsequently, the pre-processing phases are explained in the second step, as depicted in Fig. 2. Finally, a range of machine learning models was employed for the classification of facial expressions. The following subsections provide a detailed discussion of each stage in the proposed methodology.

**Fig. 3:**
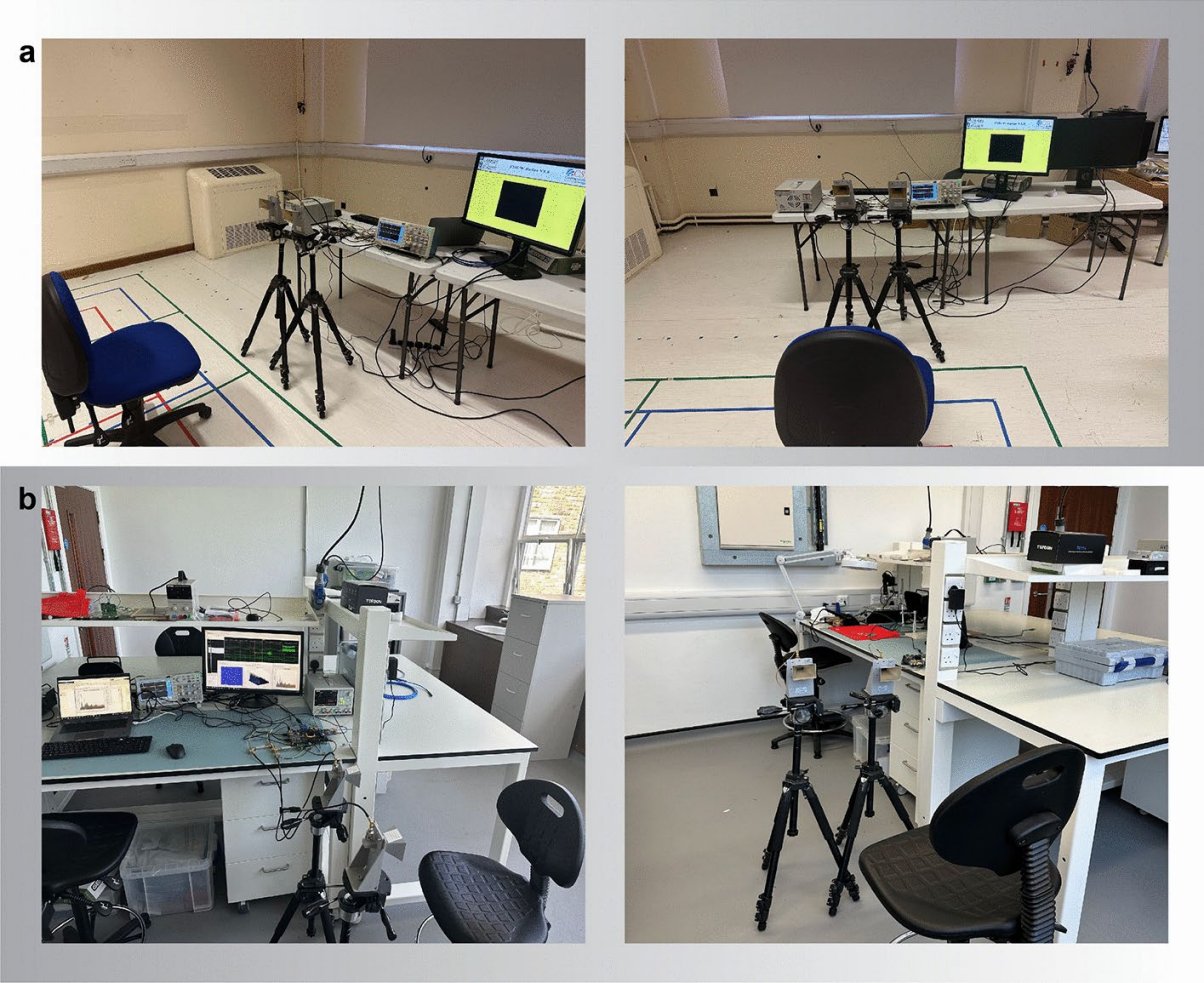
The experimental setup. (**a**) The experimental setup for room 1. (**b**) The experimental setup for room 2.

**Fig. 4:**
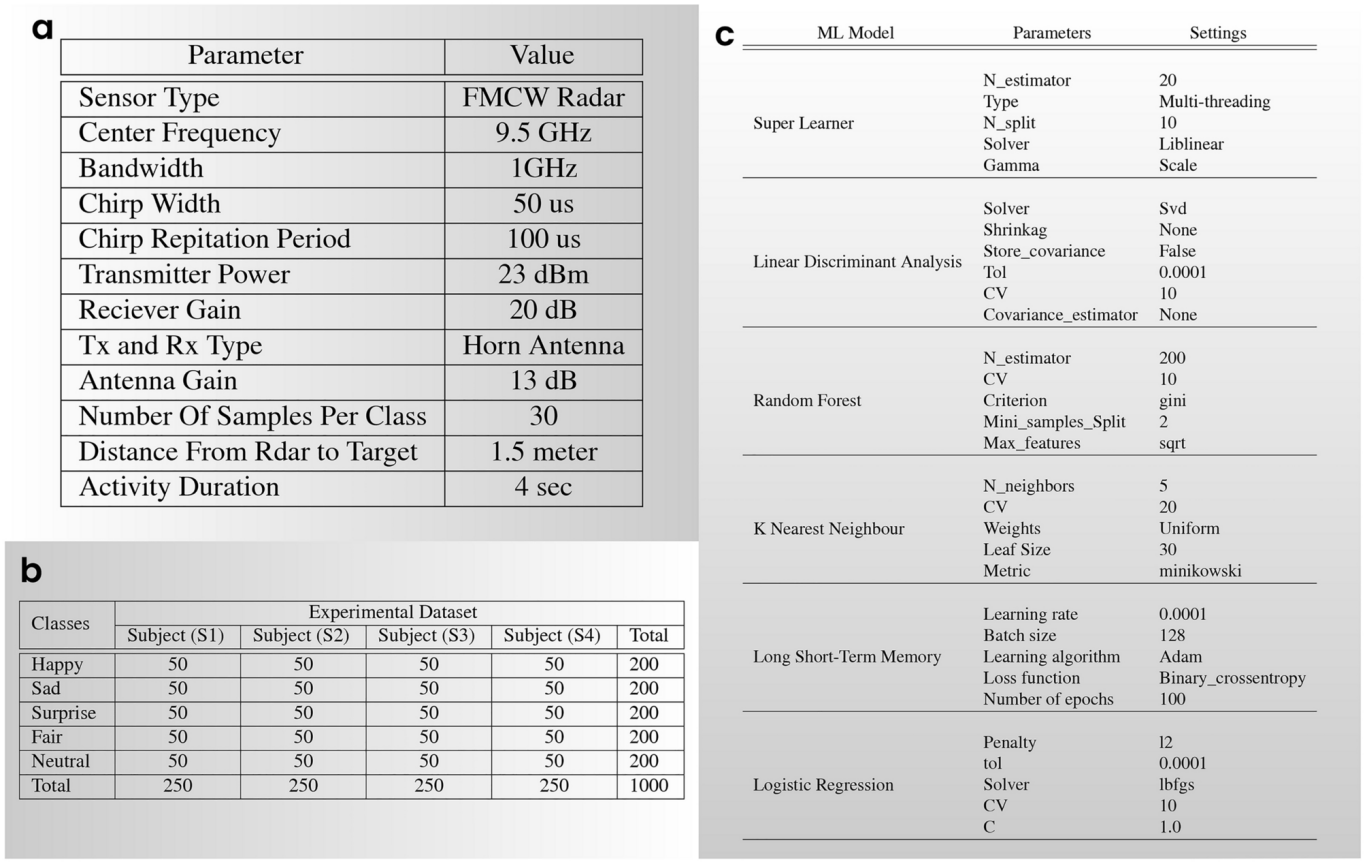
(**a**) Selected hardware and software parameter settings. (**b**) A summary of the collected data, the participant count, and the conducted activities of facial expressions. (**c**) Parameter settings for the ML models.

**Fig. 5:**
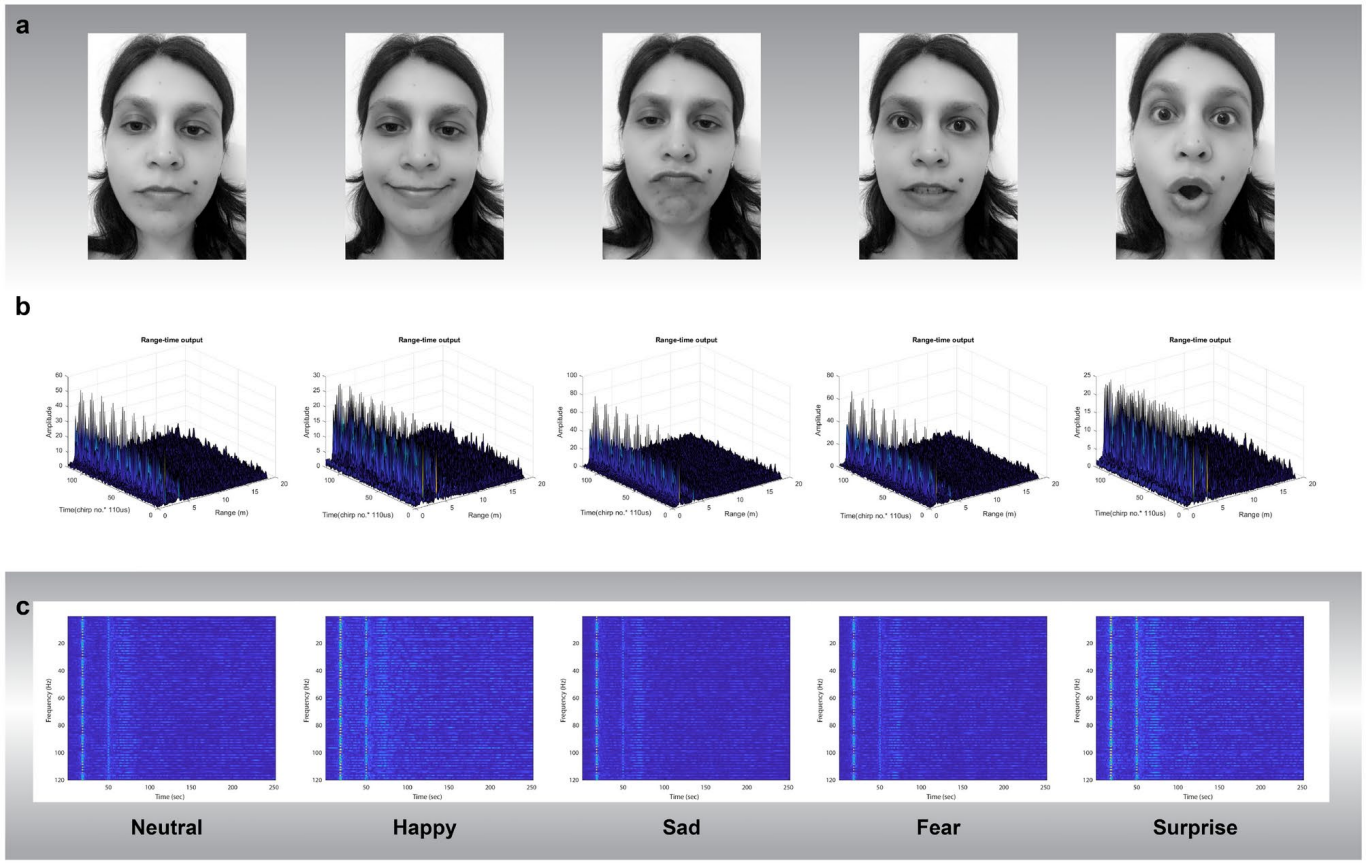
The radar signal representation of facial expression activities. (**a**) A visual representation of facial expression activities. (**b**) The range-time output of various activities (**c**) Spectogram representing various facial expression activities.

**Fig. 6:**
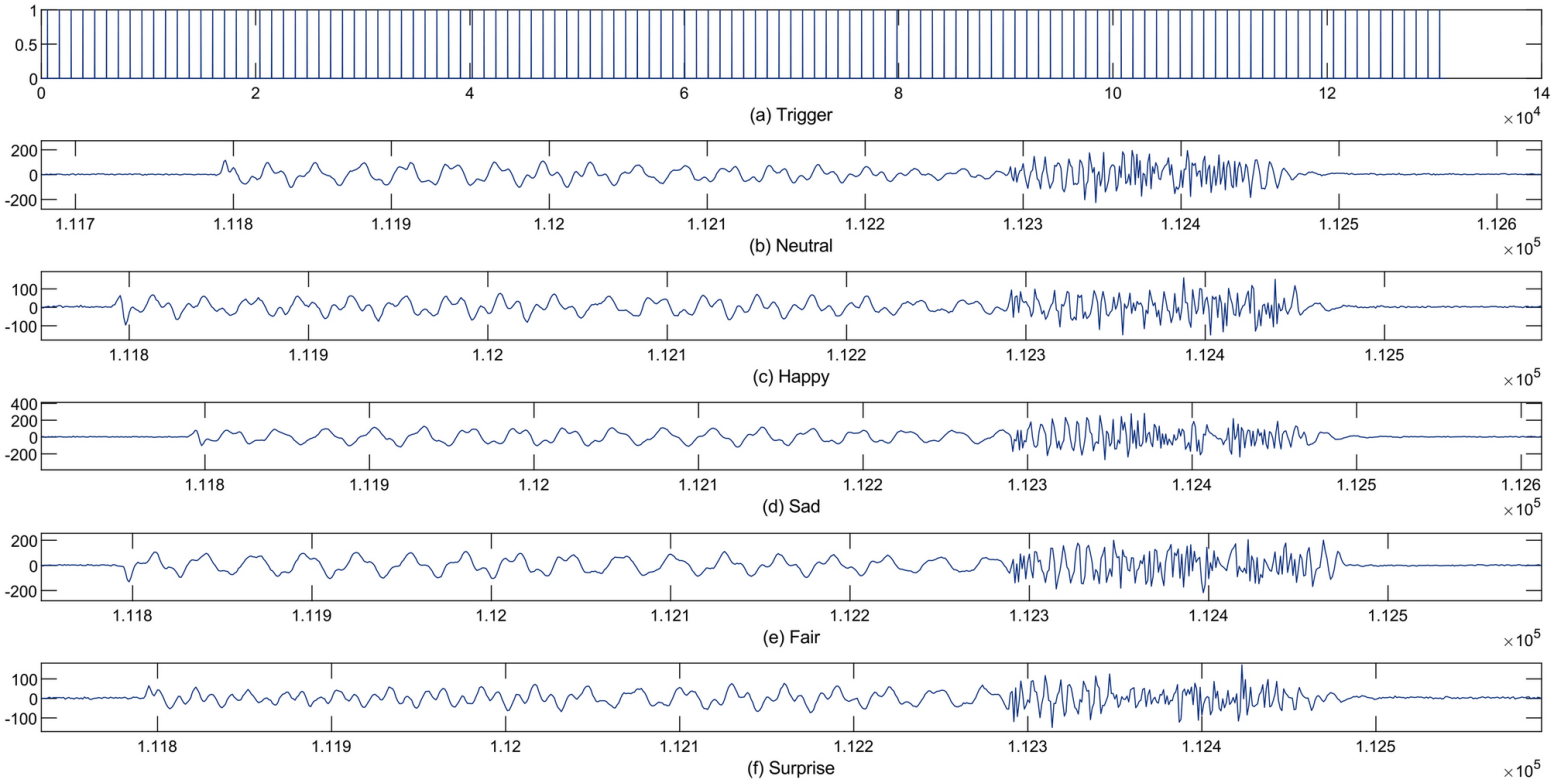
A visual representation of variation in frequency for different facial expressions (**a**) Trigger. (**b**) Neutral. (**c**) Happy. (**d**) Sad. (**e**) Fair. (**f**) Surprise.

### Experimental setup and data collection

The hardware configuration of the radar-based facial expression system is illustrated in Fig. 3. The experiments were conducted in two separate rooms, as shown in Fig. 3a,b, representing Room 1 and Room 2, respectively. The experimental setup included a Frequency Modulated Continuous Wave (FMCW) radar sensor, positioned in front of the user. The FMCW radar sensor comprised a transmitter (Tx), a receiver (Rx), and two horn antennas for transmitting and receiving, allowing for a maximum detection range of 20 meters. The key parameter settings of the radar system are outlined in Fig. 4a. During the facial expression tasks, the subject was positioned at a distance of 1.50 meters from the radar. The subject’s body remained in a natural position, with the only movements being those of the face and slight head movements, which are typical during the conversation. A single subject facial expression was recorded for each action in the study for a total of 4 s. The radar system transmitted and received the RF signal during this time period. Figure 5b presents the range time output captured during the measurement of various facial expressions. This output is utilised for signal processing and distance measurement purposes. To extract features from the radar data, the Fast Fourier Transform (FFT) is applied, generating spectrograms that illustrate the radar Doppler shift caused by facial movements as depicted in Fig. 5c. The analysis of these spectrograms reveals variations corresponding to different facial expressions due to the distinct movements of the face and mouth. The use of a trigger in a radar system is essential to ensure precise timing, synchronization, and control over the data acquisition process, as shown in Fig. 6.

### Dataset

In this phase, facial expression data was collected using FMCW radar. Figure 3 illustrates the hardware setup of the radar-based system employed for facial expression data collection. The FMCW radar sensor was equipped with two horn antennas, one for transmission (Tx) and the other for reception (Rx), enabling a maximum detection range of 20 meters. As shown in the figure, the radar sensor is placed on the table. The key parameter settings of the radar are indicated in the Fig. 4a. In order to encompass different complexity levels in the dataset, it was recorded in two different room environments. Because data collection is significantly impacted by a variety of environmental circumstances, ensuring the reliability and authenticity of the proposed system in a variety of locations. After collecting the dataset, it is proof that the system has the same behaviour in all environments. During the data collection process, the subjects maintained a neutral body position, focusing solely on facial movements. Additionally, each activity had a fixed duration of 4 s, allowing for the collection of data corresponding to a single gesture performed by an individual subject. Figure 5 provides a visual illustration of the face expression datasets. We have obtained ethical approval for the study, confirming that all research was conducted in accordance with relevant guidelines and regulations. Due to the nature of the work, informed consent was obtained from all participants for the use of identifying information and images in an online open-access publication. This approval was granted by the College of Science and Engineering at the University of Glasgow’s Research Ethics Committee, under approval numbers (300200232 and 300190109). The data collection process involved the participation of four individuals, consisting of two males and two females. The inclusion of multiple participants aimed to enhance the realism and diversity of the dataset. A total of 1000 data samples were collected during the experiment, encompassing five distinct categories across two different rooms. The details of the collected dataset are highlighted in the Fig. 4b. In each experiment, a total of 1000 data samples were collected from four participants, with 30 samples collected in each class. In particular, each participant repeated the facial expression activity of each gesture 30 times with the radar. In this way, each participant contributed to collecting 250 data samples in total for the fifth class. In each case, a total of 1000 data were categorised as fifth facial gestures, of which 800 were utilised for training and 200 for testing.

### Data pre-processing and machine learning models

The FMCW radar was used for data collection, many parameters are important for target detection in radar systems such as Tx power, Carrier frequency, antenna gain, receiver sensitivity, target cross-section area, and receiver noise. To extract facial expression accurately, first detect the target location is needed. The target range in radar systems is defined by the delay between the transmitted and received echoes. Equation 1 shows the radar range equation:

1 $$\begin{aligned} \begin{aligned} R_{max}=4\sqrt{\frac{P_{t}G_{t}G_{r}\leftthreetimes ^{2}\sigma }{(4\pi )^{3}KT_{0}BF_{n}\frac{S}{N}}} \end{aligned} \end{aligned}$$

Where $P_t$ is the transmitted power, $G_t$ is the $T_x$ antenna gain, $G_r$ is the receiver antenna gain, $\lambda$ is the wavelength, $\sigma$ is the target cross-section area, *K* is Boltzmann’s constant, *T* is the system temperature, *B* is the bandwidth, *F* is the receiver noise figure, and *S*/*N* is the signal-to-noise ratio^32,33^. Firstly, we generated of the FMCW ramp which is the fundamental step in the operation of FMCW radar systems, enabling the measurement of target range based on the frequency difference between transmitted and received echoes. After that acquired the received echoes using an Analog-to-Digital Converter (ADC) with a sampling rate of 250 MSPS (Mega Samples Per Second) and applied signal processing techniques on collected samples such as low pass filtering, target extraction, and range calculation. Signal integration technique is applied for improving SNR to enhance the signal quality and increase the probability of detection. SNR was measured of the strength of the desired signal compared to the background noise level. Downsampling technique was used to reduce the sampling rate of a signal while retaining the essential information. We did downsampling to 10 MSPS. In the data pre-processing and machine learning (ML) approach, we utilised the widely used Scikit library, which provides comprehensive data analysis tools in Python^34^. Additionally, we employed Pandas, a Python library, for parsing CSV files and converting them into data frames that can be further analyzed using Scikit-learn^35^. Labels were assigned to the first column of the data frames. In the combined dataset, produced by merging the data frames of each sample, NaN (Not a Number) values may arise due to slight mismatches in the micro-Doppler signal. To handle these NaN values, we used the SimpleImputer function from Scikit-learn to replace them with the mean of each row. It is important to note that this data cleansing process does not alter the overall data patterns. The processed data was fed into various ML algorithms following the data cleansing step. The facial expression recognition system proposed in this study is evaluated using six different machine learning (ML) methods. The evaluation parameter for assessing the system’s performance is the accuracy of correctly classifying various facial expressions. Each ML algorithm’s accuracy is assessed independently using two approaches to ensure robust analysis: (i) k-fold cross-validation and (ii) train-test split. K-fold cross-validation is a widely adopted approach in ML testing, where the dataset is divided into k groups. In this experiment, we set k to 10, resulting in the dataset being divided into 10 groups or folds. Each group is then used as a test set while the remaining groups serve as training sets. This process is repeated k times, with each group acting as the test set once. The results obtained from each group of classifications collectively represent the performance of the ML algorithms on the entire dataset. In addition to k-fold cross-validation, the train-test split technique is employed. This technique involves dividing the dataset into training and testing subsets. The training data is used to train the ML models, enabling them to learn from the provided inputs and corresponding labels. The trained algorithms are then evaluated by making predictions on the testing data based on the learned patterns. In this study, 80% of each dataset is used for training, while the remaining 20% is used for testing. Furthermore, Fig. 4c lists the parameters used to configure the ML algorithms employed in the evaluation process.

### Performance metrics for evaluating the classification model

In this study, the performance evaluation of ML models for facial expression dataset classification involves several metrics, including weighted average accuracy, precision, recall, f1-score, accuracy, and a 95% confidence interval (CI). The F1-score is a widely used metric in classification literature, serving as a measure of classification performance. It combines precision and recall, which are calculated using Eqs. (3) and (4), respectively. The F1-score, calculated using Eq. (2), provides a comprehensive evaluation of the model’s ability to balance both precision and recall in classification tasks. The overall accuracy of the combined dataset is calculated using 5 and verified the interval using 6. Where, the 95% asymptotic CI measures the statistical significance of experimental results. It represents the radius, with n = 1000 samples (20% of the dataset), and uses k as the number of standard deviations. The CI has a 95% probability of containing the true classification result. A value of k = 1.96 from the Gaussian distribution establishes this 95% confidence level.

2 $$\begin{aligned} F1-Score= & 2\frac{Precision.Recall}{Precision+Recall}\end{aligned}$$

3 $$\begin{aligned} Precision= & \frac{\sum TP}{\sum TP+\sum FP}\end{aligned}$$

4 $$\begin{aligned} Recall= & \frac{\sum TP}{\sum TP+\sum FN}\end{aligned}$$

5 $$\begin{aligned} Accuracy= & \frac{\sum (TP+TN)}{\sum (TP+FP+TN+FN)}\end{aligned}$$

6 $$\begin{aligned} Interval= & k\times \sqrt{\frac{{Accuracy\times (1-Accuracy)}}{n}} \end{aligned}$$

### Results and discussion

**Fig. 7:**
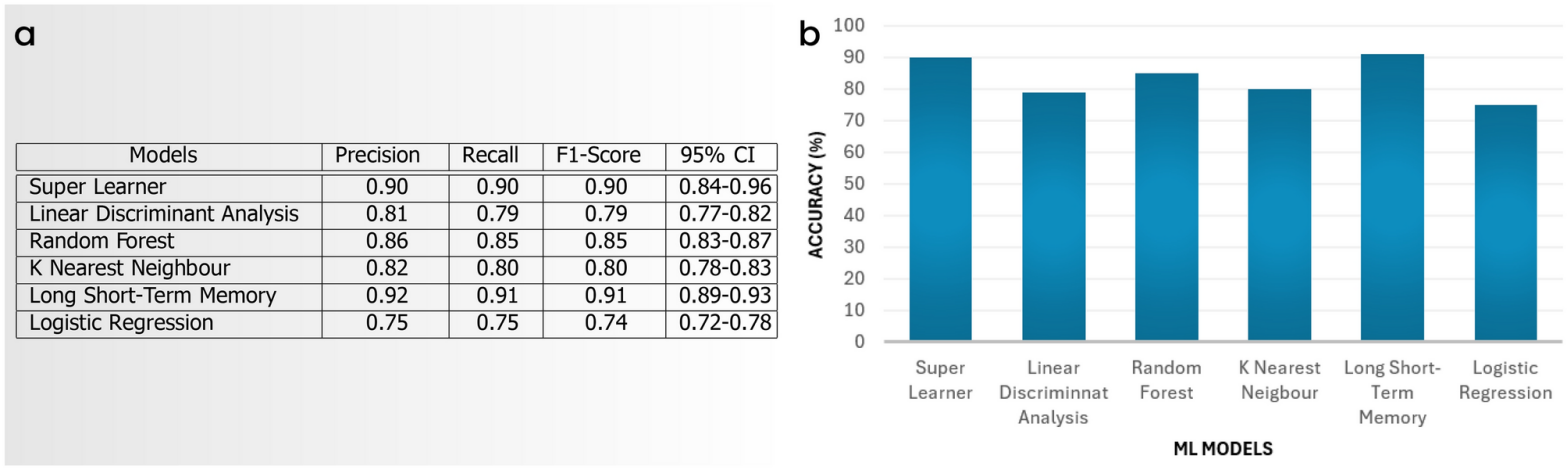
(**a**) The evaluation of the ML models on the facial expression dataset involved measuring weighted average recall, weighted average precision, weighted average F1-score, accuracy, and determining a 95% confidence interval. (**b**) The bar graph shows accuracy.

**Fig. 8:**
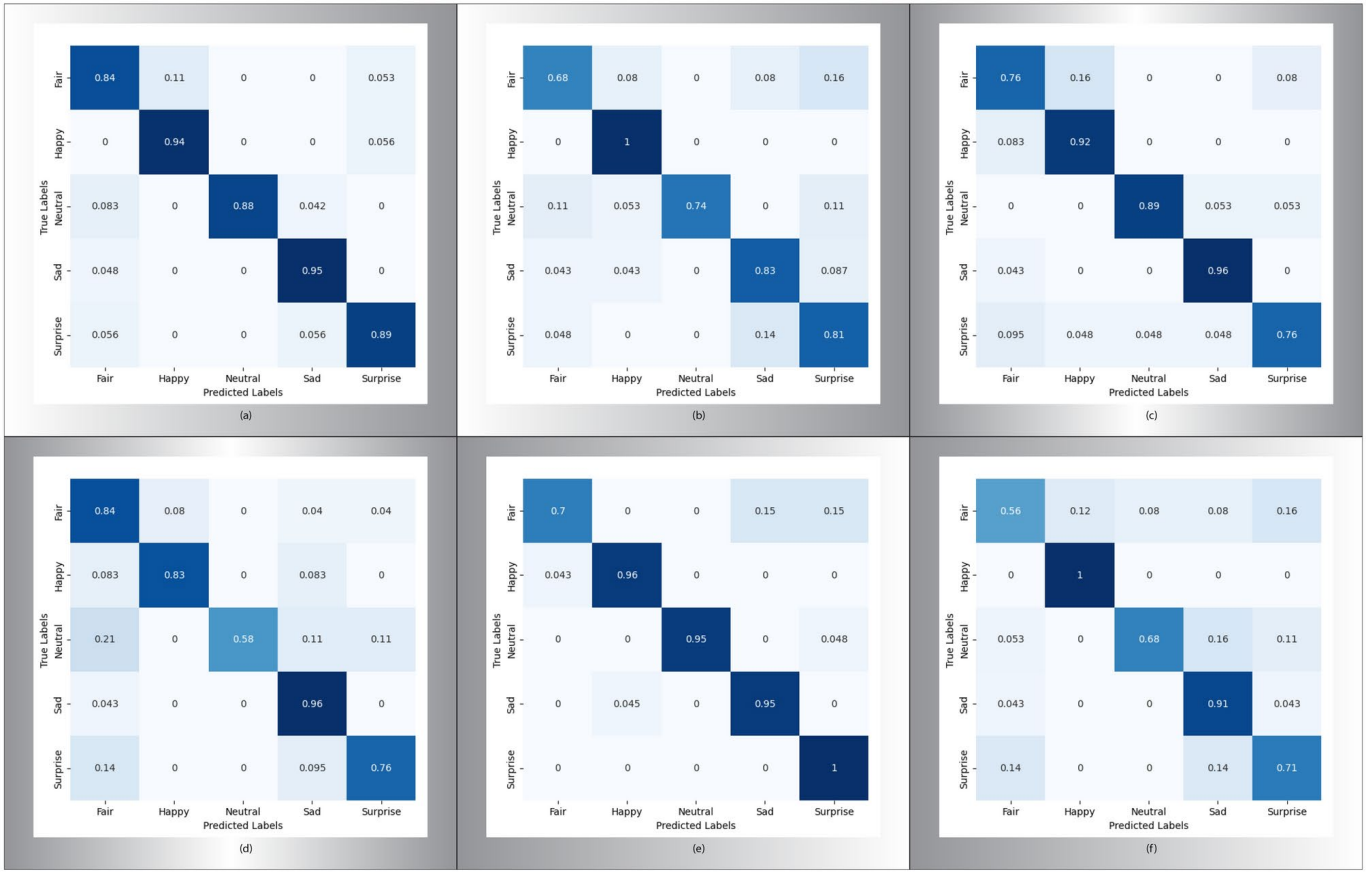
The confusion matrix of well-known ML algorithms on the combined dataset. (**a**) Super Learner. (**b**) Linear discriminant analysis. (**c**) Random Forest. (**d**) K Nearest Neighbour. (**e**) Long Short-Term Memory. (**f**) Logistic Regression.

This experimentation serves two purposes. In the first step, we introduced radar-based facial recognition, and in the next step, we compared the performance of various existing machine learning models such as Super Learner, Linear Discriminant Analysis, Random Forest, K Nearest Neighbour, Long Short-Term Memory, and Logistic Regression. We collected and analysed the performance of facial expression frameworks using different facial expression datasets such as “Neutral, Happy, Sad, Fair, and Surprise” from different genders. As a result, we performed experiments on Micro-Doppler signal data to evaluate the model’s performance. The hyper-parameter settings for all models are listed in the Fig. 4c. All of the models on the dataset have been fine-tuned. Additionally, the training and testing sets were fixed throughout all studies. The percentages of the entire data in our training and testing sets are 80% and 20%, respectively. Figure 7a shows the outcomes of studies with various facial expression structures in terms of precision, recall, F1-score, and confidence interval. Figure 7b presents a bar graph illustrating accuracy, which aids in decision-making and comparisons. Figure 8 shows the confusion matrix of all proposed models on collected datasets. Overall, better outcomes were obtained for the combined and individual datasets using all models.

In the super learner algorithm, the combined dataset includes males and females. We got a high classification accuracy of 90% with precision, recall, and F1-score and accurate interval, which are shown in Fig. 8a. All the classes are correctly classified except “Fair” because 11% of expressions are similar to “Happy”.

Similarly, Linear Discriminant Analysis is well-performed on the combined dataset with 80% accuracy, precision, recall, f1-score and valid interval which are shown in Fig. 8b. All the classes are correctly classified except “Fair” which has been misclassified with “Happy”, “Sad”, and “Surprise” with a ratio of 0.08, 0.08, and 0.16.

Using Random Forest, the combined dataset includes males and females. We got a high classification accuracy of 85% with precision, recall, f1-score, and accurate interval which are shown in Fig. 8c. All the classes are correctly classified except “fair” and “surprise”. The “Fair” has similarities with “Happy” with a ratio of 0.16. Here again, “surprise” has similarities with “fair”, “happy”, “neutral”, and “sad” with ratios of 0.095, 0.048, 0.048, and 0.048.

In the case of the K Nearest Neighbour algorithm, we got a high classification accuracy of 80%, precision, recall, f1-score, and interval, which are shown in Fig. 8d. Except for “surprise” all classes are correctly classified because it has similarity with “fair” and “sad” with a ratio of 0.14 and 0.095.

Using the Long Short-Term Memory algorithm on the combined dataset, we got high classification accuracy of 91%, precision, recall, f1-score, and interval, which are shown in Fig. 8e. All classes are correctly classified except “Fair” which has been misclassified with “sad” and “surprise” with ratios of 0.15 and 0.15.

Logistic Regression, the combined dataset includes males and females. We got a high classification accuracy of 75% with precision, recall, and F1-score and an accurate interval, which are shown in Fig. 8f. All the classes are correctly classified except “fair” because it has similarities with “happy”, “neutral”, “sad”, and “surprise” with ratios of 0.12, 0.08, 0.08, and 0.16.

## Conclusion and future work

This paper presents a contactless and privacy-preserving facial recognition framework. The diverse dataset is taken from different users in the form of micro-doppler signals and fed into well-known machine learning models. The collected data consists of five different classes: neutral, happy, sad, fair, and surprise. The experiment included four participants, two male and two female, aged 20 to 40 years. The micro-Doppler data is fed into various machine learning models, including Super Learner, Linear Discriminant Analysis, Random Forest, K Nearest Neighbour, Long Short-Term Memory, and Logistic Regression. The face movements were mostly classified correctly, achieving a 100% accuracy rate. Among the models tested, the Long Short-Term Memory algorithm performed the best, with an overall accuracy of 91% for all five classes. Future research will focus on improving radar signal processing to enhance accuracy under various conditions. We plan to explore more advanced machine learning models to better differentiate between subtle facial expressions and increase the robustness of emotion recognition. Additionally, we will direct our research toward integrating our radar system with other sensing modalities, such as infrared or thermal imaging, to provide a more comprehensive understanding of facial expressions. We will also investigate ways to make the technology more adaptable to diverse age groups by incorporating a wider range of facial data into our training sets.

## Data Availability

The datasets utilised in the current study are available from the corresponding author upon reasonable request at *qammer.abbasi@glasgow.ac.uk*.

## Additional information

Correspondence and requests for materials should be addressed to H.H., or Q.H.A.
